# ENDOCRINE DISRUPTORS: Estrogens in a Bottle?

**DOI:** 10.1289/ehp.117-a241

**Published:** 2009-06

**Authors:** Julia R. Barrett

Much of our exposure to endocrine disruptors occurs through what we eat and drink—in some cases, chemicals such as plasticizers may have migrated from food or beverage packaging. The possibility that these chemicals end up in commonly consumed beverages was the focus of two recent European studies that found evidence of estrogenic activity in mineral water. Both studies focused on the estrogenic potential of mineral water bottled in polyethylene terephthalate (PET) plastic, the material constituting most convenience−size beverage bottles sold in the United States today.

In the first study, published in the March 2009 *International Journal of Hygiene and Environmental Health*, a recombinant yeast−based *in vitro* assay was used to assess estrogenic activity in 30 PET−bottled mineral water samples. Ninety percent of the samples tested negative for estrogenic activity. Of the remaining samples, most showed measurements corresponding to a range of 14–23 ng/L estradiol equivalents—similar to the estrogen burden posed by treated drinking water derived from groundwater and river water (15 and 17 ng/L estradiol equivalents, respectively).

Of the estrogen−positive samples, authors Barbara Pinto and Daniela Reali, investigators in the University of Pisa Department of Experimental Pathology, say the water may have been contaminated at its source, during processing, or after bottling. They cite several studies showing that suboptimal storage conditions—such as prolonged exposure to sunlight and high temperatures—can cause leaching of chemicals from PET bottles into fluid contents, and point out that “cell toxicity was observed for water samples of the same lot of three different brands purchased from the same retailer.”

Estrogenic activity in PET−bottled mineral water was also observed by graduate student Martin Wagner and chairman Jörg Oehlmann of the Department of Aquatic Ecotoxicology at Johann Wolfgang Goethe University. Using a similar but more sensitive yeast−based estrogen screen, the researchers tested 20 brands of mineral water packaged in PET, glass, or coated paperboard. Elevated estrogenic activity was measured in 12 of 20 brands of mineral water, including 78% of those bottled in PET and 33% of those bottled in glass. However, multiuse PET bottles (which are intended to be reused several times) showed lower estrogenicity than bottles meant for one−time use—and were even lower than glass bottles from the same mineral water source.

This study, published online 10 March 2009 in *Environmental Science and Pollution Research*, also included experiments in which mud snails (*Potamopyrgus antipodarum*), an organism that is highly sensitive to estrogens, were raised in glass and PET bottles. The findings mirrored those from the yeast−based assay, but with one interesting exception: A PET sample that showed minimal response in the yeast assay induced one of the more significant results in the mud snail assay.

The disparity implies bottled water may contain a mix of compounds. “Perhaps the snails were reacting to, for example, anti−androgens coming from these plastic bottles. We would have missed these *in vitro* because we only looked for [estrogen receptor] ligands,” Wagner says. Although he and Oehlmann also noted several points at which contamination could have occurred during water processing, Wagner says the snail data led them to conclude that at least some contamination arose from the PET bottles: “Because the snail experiment did not use mineral water but rather a defined culture medium for snails, which was the same in all bottles, the estrogenic effect in the snails could only have come from the packaging material.”

This conclusion has been strongly discounted by several industry groups, including the PET Resin Association (PETRA). “It has been demonstrated through extensive studies that PET meets all established safety standards for use in food and beverage packaging and has been safely used for that purpose for decades,” says Ralph Vasami, executive director of PETRA. The organization also emphasizes that PET destined for food and beverage containers does not contain bisphenol A or orthophthalates, both of which have been heavily scrutinized as endocrine disruptors.

Still, we should think about the components of PET plastic in terms of potential leaching of products that have biological activity, says Kris Thayer, a staff scientist at the National Toxicology Program’s Center for the Evaluation of Risks to Human Reproduction, in response to the Italian and German studies. “If people are moving away from polycarbon−ate plastics [due to bisphenol A concerns], what do they use instead? When we consider alternative plastics, we need to be sure they are characterized,” she says. Part of the characterization process entails finding out which compounds, if any, leach from the plastic.

Neither of the European studies can be used to deduce anything about potential human health effects of drinking PET−bottled beverages. However, if PET bottles do leach endocrine−disrupting chemicals into the beverages they contain, it could represent a significant source of exposure for many people. According to figures from the Beverage Marketing Corporation published in the April/ May 2009 issue of *Bottled Water Reporter*, Americans drank 108 L of bottled water per person in 2007, while per−capita Italian consumption reached 204 L.

## Figures and Tables

**Figure:**
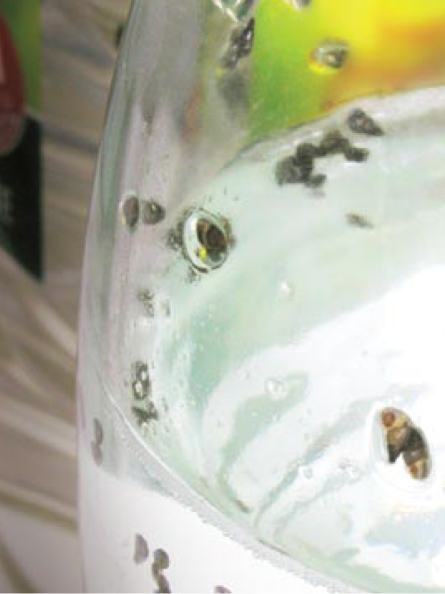
PET-housed snails produced up to twice as many embryos as glass-housed snails.

